# Dietary Copper Intake and Its Association With Telomere Length: A Population Based Study

**DOI:** 10.3389/fendo.2018.00404

**Published:** 2018-07-30

**Authors:** Zhu Lin, Hongmei Gao, Bing Wang, Yongqiang Wang

**Affiliations:** Department of Intensive Care Unit, Tianjin First Central Hospital, Tianjin, China

**Keywords:** Telomere, copper, NHANES, epidemiology, nutrition

## Abstract

**Background:** Telomere is regarded as the fundamental aspect of cellular aging and copper is recognized as one of the most essential trace elements. The role of dietary copper intake in telomere length maintenance is seldom examined. This study aims to investigate if telomere length is to be associated with daily dietary copper intake.

**Methods:** We used epidemiological data from a large national population-based health and nutrition survey. Dietary intake was assessed during the 24–h period before the interview date when blood sample was collected. Telomere length was measured from blood leukocyte using PCR method. The relationship between telomere length and dietary copper intake was assessed using multivariable linear regression models. We also examined if obesity, measured by body mass index, could modify the observed association.

**Results:** There are 7,324 participants had both leukocyte telomere length measured and dietary copper intake assessed, around 48.0% of them were men. Telomere length was longer in women than that in men (1.05 ± 0.26 vs. 1.00 ± 0.26 T/S ratio), while dietary copper intake was less in women than that in men (1.12 ± 0.80 vs. 1.51 ± 1.61 mg). After controlling for age, sex, ethnicity, physical activity, current smoking status, hypertension, cardiovascular diseases, and body mass index in the multivariable linear regression models, one unit increase of log-transformed dietary copper intake was significantly associated with longer telomere length (β = 0.02, 95% confidence interval: 0.01, 0.04). We did not find a significant sex difference for this association.

**Conclusions:** Dietary copper intake was significantly associated telomere length.The role of copper in human health might be involved in biological aging process.

## Introduction

Copper is a kind of essential trace elements in human beings as well as other species. As a transition trace element, it is a significant component for many enzymes. Besides its essential role involving in iron metabolism (1), it also participates in a number of biological processes, including oxidative stress (2), immunological function (3), and neurotransmitter synthesis (4). Dietary copper deficiency has been shown to lead to adverse human health outcomes throughout the whole life course (5). Copper deficiency may result in impaired cardiovascular system development, bone malformation (6), dyslipidemia (7), and continuous neurologic abnormalities for both infants and adult (8, 9).

Telomere length is involved in biological aging (10). Telomere length shortening was associated with cardiovascular disease (11), neurodegenerative disorders (12–14), and other metabolic diseases (15–17). Studying the determinants of telomere shortening is of paramount importance in disentangling the pathophysiology of major chronic diseases. Previous studies have shown that some nutrients were related to telomere length shortening and maintenance (18), and found superoxide dismutase could slow telomere length shortening in human fibroblasts (19) and antioxidant therapy could also attenuate the reduction of telomerase activity in superoxide dismutase-deficient mice (20). While copper is a major component of superoxide dismutase and diet is one of the main sources for copper, it is thus natural to hypothesize that dietary copper intake could be related to leukocyte telomere length. However, this association of dietary copper intake and leukocyte telomere length has rarely been examined. In this study, we tested this association using a large population based survey data, the National Health and Nutrition Examination Survey (NHANES).

## Methods

### Study design and participants

The National Center for Health Statistics of the Centers for Disease Control and Prevention has been collecting the NHANES data for decades. Written informed consent was obtained from all study participants. The survey plan and study protocol were approved by the Research Ethics Review Board at the National Center for Health Statistics. The data from NHANES 1999–2000 and 2001–2002 were combined for these analyses because leukocyte telomere length was assessed in these two data collection cycles. The final analytic sample included 7,832 participants who had their 24–h dietary intake data assessed and had telomere length measured.

### Dietary copper intake

NHANES survey participants took part in an in-person household interview and underwent a health examination in a mobile examination center, where the participants also provided a 24–h dietary recall data collection session. All study participants were asked to provide data of dietary intake during the 24–h period before the interview date when blood sample was collected. The dietary data collection was performed using the NHANES computer-assisted dietary interview system in the mobile examination center. Based on the study protocol, all participants were randomized to either a morning or an afternoon/evening exam sessions for the data collection. The completed interview data were electronically transferred to the data monitor center. And then data were entered into the University of Texas Food Intake Analysis System and USDA Survey Nutrients Database for coding dietary copper intake values.

### Telomere length assessment

Blood samples were used for leukocyte telomere length assessment. The telomere length assessment experiments were conducted in the laboratory of Prof. Elizabeth Blackburn at the University of California, San Francisco, US. The quantitative polymerase chain reaction (qPCR) method was employed to measure leukocyte telomere length, which was a relative measurement in relation to a standard reference (coded in a T/S ratio scale). The detailed methods used for telomere assessment as described elsewhere previously (21–23). In brief, the single-copy gene used for this PCR experiment (human beta-globin) was *hbg1* [5′GCTTCTGACACAACTGTGTTCACTAGC-3′] (23). Blood samples were assayed three times on three different days on duplicate plate wells, generating six data points that were used for the standard curve. Quality control was performed as follows. If the assay had eight or more invalid control wells, then they were considered invalid and were excluded from further analysis (<1% of experiments failed this criterion). The inter-assay coefficient of variation was 4.4%. The extreme T/S ratio values in the data set were considered as potential outliers. Then the mean of the T/S ratio value was calculated by excluding the potential outliers (24).

### Statistical analysis

We performed all statistical analyses using SAS (version 9.4, SAS Institute Inc, Cary, NC) software. We used sample weights to account for planned oversampling of some groups. We presented continuous variables as mean ± standard deviations, and categorical variables as number and proportions. Dietary copper intake was log-transformed to achieve a normal distribution. Dietary copper intake was used as both a continuous variable and a categorical variable in the regression models. We constructed three linear regression models to assess the association between dietary copper intake and leukocyte telomere length. The first model was a crude estimate of this association, while the second model was further adjusted for age, sex, and ethnicity. The third model was additionally controlled for physical activity, smoking status, hypertension, cardiovascular diseases, and body mass index. We also examined if the association differed between men and women by including an interaction term of sex and dietary copper intake. These models were specified as follows:

*Model 1:* $Telomere\;length=\beta_{1}^{*}copper\;intake$
*Model 2:* $Telomere\;length=\beta_{1}^{*}copper\;intake+\beta_{2}^{*}Age+\beta_{3}^{*}sex+\beta_{4}^{*}ethnicity$
*Model 3:* $Telomere\;length=\beta_{1}^{*}copper\;intake+\beta_{2}^{*}Age+\beta_{3}^{*}sex+\beta_{4}^{*}ethnicity+\beta_{5}^{*}BMI+\beta_{6}^{*}physical\;activity+\beta_{7}^{*}smoking\;status+\beta_{8}^{*}hypertension+\beta_{9}^{*}cardiovascular\;diseases$

## Results

**Figure 1:**
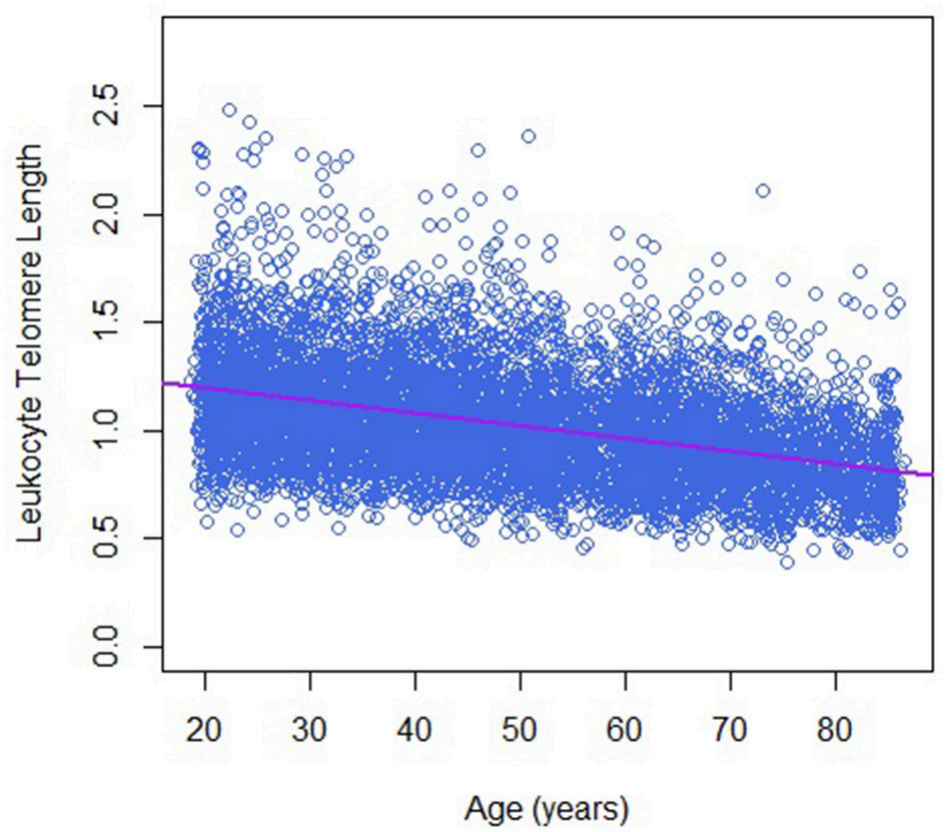
Telomere length and age in this study population.

Table 1 describes the basic characteristics of study participants by sex. There are 7,324 participants in the final analytic sample; around 48.0% of them were men. The average age was 50.2 ± 18.1 years for men and 47.9 ± 18.8 years for women. Leukocyte telomere length decreased with increasing age (−0.06 T/S ratio per year increase in age, Figure 1), and was longer in women than that in men (1.05 ± 0.26 vs. 1.00 ± 0.26 T/S ratio), while dietary copper intake was less in women that that in men (1.12 ± 0.80 vs. 1.51 ± 1.61 mg).

**Table 1:** Basic Characteristics of Study Participants.

**Variables**	**Men (*n* = 3520)**	**Women = 3804)**
Age (years)	50.2 ± 18.1	47.9 ± 18.8
Weight (kg)	85.0 ± 18.5	74.5 ± 18.9
Height (cm)	174.2 ± 7.9	160.8 ± 7.3
BMI (kg/m2)	27.9 ± 5.4	28.8 ± 6.8
Telomere (T/S ratio)	1.00 ± 0.26	1.05 ± 0.26
Copper Intake (mg)	1.51 ± 1.61	1.12 ± 0.80
Log-transformed copper intake	0.23 ± 0.59	−0.03 ± 0.53
**ETHNICITY, *n*(%)**		
Mexican American	845 (24.01)	927 (24.37)
Other Hispanic	175 (4.97)	216 (5.68)
Non-Hispanic White	1819 (51.68)	1896 (49.84)
Non-Hispanic Black	584 (16.59)	645 (16.96)
Other Race	97 (2.76)	120 (3.15)
Physical activity, *n*(%)	1222 (34.7)	934 (24.6)
Current Smoking, *n*(%)	717 (20.4)	574 (15.1)
Hypertension, *n*(%)	999 (28.4)	1157 (30.4)
Cardiovascular diseases*, n*(%)	430 (12.2)	306 (8.0)

*Mean and standard deviation were presented for continuous variables, number and proportion were presented for categorical variables*.

Table 2 presents the association between telomere length and dietary copper intake. Compared with participants with the lowest dietary copper intake in the first quantile, telomere length was longer in the fourth quantile of dietary copper intake in the first model (β = 0.05, 95% confidence interval: 0.02, 0.07). After controlling for age, sex, ethnicity, and body mass index in the multiple linear regression models, the association magnitude decreased slightly but remained significant in the third model (β = 0.04, 95% confidence interval: 0.01, 0.06). When dietary copper intake was modeled as a continuous variable, one unit increase of log-transformed dietary copper intake was significantly associated with longer telomere length (β = 0.02, 95% confidence interval: 0.01, 0.04). No significant sex difference was observed for this association.

**Table 2:** Association of Telomere Length with Copper Intake, β(95% CI).

**Copper Intake**	**Model 1**	**Model 2**	**Model 3**
Quantile 1	reference	reference	reference
Quantile 2	0.01 (−0.02, 0.03)	0.01 (−0.01, 0.03)	0.01 (−0.01, 0.03)
Quantile 3	0.03 (0.01, 0.05)	0.02 (0.003, 0.04)	0.02 (0.002, 0.04)
Quantile 4	0.05 (0.02, 0.07)	0.04 (0.01, 0.06)	0.03 (0.01, 0.06)
Continuous	0.03 (0.02, 0.04)	0.02 (0.01, 0.04)	0.02 (0.01, 0.03)

*Model 1 included only the exposure variable, log-transformed copper intake, Model 2 was additionally adjusted for age, sex, and ethnicity. Model 3 was further adjusted for BMI, physical activity, current smoking, hypertension, and cardiovascular disease. The values represent β(95% CI)*.

## Discussion

In the present study, we investigated the association of TL with dietary copper intake in a large population-based health and nutrition survey using the NHANES data, and found that dietary copper intake was association with longer telomere length. This reported association remained significant after controlling for various potential covariates including age, sex, ethnicity, but was attenuated only slightly with body mass index adjustment, suggesting that dietary copper might influence telomere through biological pathways beyond obesity. We did not find significant sex differences of these associations.

Dietary copper has been reported to be predictive of metabolic related disorders in both observational population studies and animal experiments (25, 26). One study consisting of more than one thousand participants found dietary copper intake was inversely associated with hypertension, blood pressure, glycemic traits, lipid fraction levels, and uric acid (25). Another study tested the association of dietary copper intake with cognitive ability and found that high dietary intake of copper in conjunction with a diet high in saturated and trans fatty acid may be associated with accelerated cognitive decline in the elderly population (27). Because telomere length is regarded as the fundamental molecular aspects of biological aging, it is interesting to examine if telomere is correlated to dietary copper intake. However, this association has seldom been investigated. Only a few studies reported a significant association between trace element and telomere length (28, 29), one of them used human hepatocytes and hematoma cell lines and the other used data from a cross-sectional survey.

Previous studies have also found that obesity was significantly associated with leukocyte telomere length (30). Obese people had shorter telomere length compared with their lean peers. Likewise, obesity is one of the most important risk factors for chronic diseases such as cardiovascular and neurodegenerative diseases (31, 32). It is also closely related to dietary nutrients intake (33). Thus, we hypothesized obesity could confound the association of leukocyte telomere length and dietary copper intake. In this study, we tested this hypothesis and found the results did not change very much. When additionally adjusting for body mass index, the magnitudes of these associations were attenuated only slightly. This means the observed telomere length and dietary copper intake relationship was independent of obesity. In the present study, we found women had longer telomere length than men. This finding is in line with previous studies (34).

The relationship between copper and telomere are not quite clear. Several potential biological mechanisms might explain the observed association. Copper is known to be an essential nutrient and functions as a key element for enzymes. One of the mostly studies enzyme related to copper is superoxide dismutase (35), which helps cells to break down potentially harmful reactive oxygen (36) that could accelerate telomere shortening (37). Besides that, pharmacological level of copper has been found to be able to induce the immune and antioxidant mechanisms *in vivo* (38). Enhanced immunological function could also slow down telomere shortening induced by infection (39).

Several strengths deserve to be noted for the present study. First, telomere length was measured in a lab using well-established methods. Second, the study samples were very large and participants were representative of the national US population from which study participants were randomly invited to NHANES. The large sample size was also representative of the US population with a broad age interval. These results could thus be generalized. Lastly, statistical regression models took into account of multiple potential confounders including age, sex, and body mass index. The results showed that the association between leukocyte telomere lengths with dietary copper intake was independent of these potential confounders. However, several limitations should also be acknowledged. This study used a national survey that was cross-sectional by design. The nature of this study design makes causal inference based on our analyses difficult. The association between dietary copper intake and telomere length does not imply increasing dietary copper could increase telomere length. Residual confounding could still bias this association and should be considered by other study design or advanced statistical analyses. Additionally, the present study did not include genetic variants or telomerase activity assessment. No information was available regarding the cell types which might affect telomere length assessment. The circulating copper was also unavailable from NHANES. Examining these genetic biomarkers, cell types, and blood or urine copper could probably provide more valuable evidence to assess the present results.

In summary, this study demonstrates that dietary copper intake significantly correlates with telomere length in participants of NHANES. Though our findings are derived in a cross-sectional survey, the discovery of dietary copper intake as a prognostic determinant of telomere length, independent of known obesity, may enhance our understanding of disease diagnosis or prognosis and shed light on the disease mechanisms underlying the association between biological aging and other metabolic disorders.

## Ethics statement

This study was carried out in accordance with the recommendations of NHANES committee with written informed consent from all subjects. All subjects gave written informed consent in accordance with the Declaration of Helsinki. The protocol was approved by the NHANES committee.

## Author contributions

YW and ZL designed the study. All authors drafted and revised the manuscript.

### Conflict of interest statement

The authors declare that the research was conducted in the absence of any commercial or financial relationships that could be construed as a potential conflict of interest.
